# Stimulated Raman histology for histological evaluation of oral squamous cell carcinoma

**DOI:** 10.1007/s00784-023-05098-9

**Published:** 2023-06-22

**Authors:** David Steybe, Philipp Poxleitner, Marc C. Metzger, René Rothweiler, Jürgen Beck, Jakob Straehle, Kirstin Vach, Andreas Weber, Kathrin Enderle-Ammour, Martin Werner, Rainer Schmelzeisen, Peter Bronsert

**Affiliations:** 1 Department of Oral and Maxillofacial Surgery, Medical Center – University of Freiburg, Faculty of Medicine, University of Freiburg, Freiburg, Germany; 2 Center for Advanced Surgical Tissue Analysis (CAST), University of Freiburg, Freiburg, Germany; 3 Department of Oral and Maxillofacial Surgery and Facial Plastic Surgery, LMU University Hospital, LMU Munich, Lindwurmstrasse 2a, 80337 Munich, Germany; 4 Department of Neurosurgery, Medical Center – University of Freiburg, Faculty of Medicine, University of Freiburg, Freiburg, Germany; 5 Institute of Medical Biometry and Statistics, Faculty of Medicine and Medical Center, University of Freiburg, Freiburg, Germany; 6 Institute for Surgical Pathology, Medical Center – University of Freiburg, Faculty of Medicine, University of Freiburg, Freiburg, Germany; 7 Tumorbank Comprehensive Cancer Center Freiburg, Medical Center, University of Freiburg, Freiburg, Germany; 8 Core Facility for Histopathology and Digital Pathology, Medical Center, University of Freiburg, Freiburg, Germany

**Keywords:** Oral squamous cell carcinoma, Pathology, Raman spectroscopy, Microscopy, Surgical margins

## Abstract

**Objectives:**

To investigate whether in patients undergoing surgery for oral squamous cell carcinoma, stimulated Raman histology (SRH), in comparison with H&E-stained frozen sections, can provide accurate diagnoses regarding neoplastic tissue and sub-classification of non-neoplastic tissues.

**Materials and methods:**

SRH, a technology based on Raman scattering, was applied to generate digital histopathologic images of 80 tissue samples obtained from 8 oral squamous cell carcinoma (OSCC) patients. Conventional H&E-stained frozen sections were then obtained from all 80 samples. All images/sections (SRH and H&E) were analyzed for squamous cell carcinoma, normal mucosa, connective tissue, muscle tissue, adipose tissue, salivary gland tissue, lymphatic tissue, and inflammatory cells. Agreement between SRH and H&E was evaluated by calculating Cohen’s kappa. Accuracy of SRH compared to H&E was quantified by calculating sensitivity, specificity, positive predictive value (PPV), and negative predictive value (NPV) as well as area under the receiver operating characteristic curve (AUC).

**Results:**

Thirty-six of 80 samples were classified as OSCC by H&E-based diagnosis. Regarding the differentiation between neoplastic and non-neoplastic tissue, high agreement between H&E and SRH (kappa: 0.880) and high accuracy of SRH (sensitivity: 100%; specificity: 90.91%; PPV: 90.00%, NPV: 100%; AUC: 0.954) were demonstrated. For sub-classification of non-neoplastic tissues, SRH performance was dependent on the type of tissue, with high agreement and accuracy for normal mucosa, muscle tissue, and salivary glands.

**Conclusion:**

SRH provides high accuracy in discriminating neoplastic and non-neoplastic tissues. Regarding sub-classification of non-neoplastic tissues in OSCC patients, accuracy varies depending on the type of tissue examined.

**Clinical relevance:**

This study demonstrates the potential of SRH for intraoperative imaging of fresh, unprocessed tissue specimens from OSCC patients without the need for sectioning or staining.

## Introduction

The oral cavity is among the frequent sites of malignant cancer development. In the 2020 Global Cancer Statistics, 377,713 new cases and 177,757 deaths related to cancers of this location were reported [1], with squamous cell carcinoma accounting for > 90% of cases [2]. In the multimodal treatment of OSCC, surgical resection is an important pillar. In this context, intraoperative histopathological differentiation between neoplastic and non-neoplastic tissue is of great importance to ensure complete tumor resection while striving to preserve structures not affected by the tumor to ensure good postoperative function and quality of life [3]. The current standard for intraoperative differentiation between neoplastic and non-neoplastic tissue is based on H&E-stained frozen sections; however, this approach is time and labor intensive and requires a well-equipped histopathology laboratory with experienced technical assistants. In order to bypass traditional histopathologic sectioning and staining, a number of novel technologies have been presented in the past. One of these technologies is Raman spectroscopy, which is based on the inelastic scattering of photons and allows the characterization of the chemical composition of tissue samples by analyzing the natural vibrational properties of molecules [4]. Diagnosis of OSCC by analysis of Raman spectra obtained from serum, saliva, or tissue samples has been studied by a number of investigators [5–8]. Generally, in these studies, point measurements were performed at a relatively low number of different positions of the samples in order to investigate the molecular composition of the sample by analyzing the respective Raman spectra.

Aiming at providing background-free and readily interpretable chemical contrast, Freudiger et al. introduced stimulated Raman scattering microscopy in 2008, laying the foundation for the use of Raman scattering to obtain high-resolution microscopic images of unprocessed tissue samples [9]. Further refinement of this technology led to the NIO Laser Imaging System (NIO Laser Imaging System, Invenio Imaging Inc., Santa Clara, CA, USA), a mobile, stand-alone clinical SRS microscope that allows this technique to be performed in a clinical setting without the need for processing, staining, or labeling tissue specimens and thus without the need for a histopathology laboratory and technical assistants [7, 10].

The system applies two Raman shifts (2845 cm^−1^ which corresponds to CH2 bonds that are abundant in lipids; 2940 cm^−1^ which corresponds to CH3 bonds that predominate in proteins and DNA) to generate images with contrast suitable for visual tissue analysis. With the aim of facilitating visual evaluation, the acquired data are further processed to generate images reminiscent of conventional H&E images. To date, this method — termed stimulated Raman histology (SRH) — has mainly been applied in the field of neurosurgery with remarkable agreement with conventional H&E microscopy [10, 11].

The aim of the present study was to investigate the applicability of SRH for the detection of neoplastic tissue in OSCC patients, which would provide a basis for the use of this technology in the intraoperative evaluation of surgical margins. In addition, the potential of SRH for sub-classification of non-neoplastic tissue in samples derived from OSCC patients was investigated.

## Materials and methods

This prospective study was approved by the Ethics Committee of the University of Freiburg (reference: 22-1037). Written informed consent was obtained from all patients before inclusion. Inclusion criteria for the study were as follows: legal age (> 18 years), biopsy proven OSCC without neoadjuvant radio- (RT)/radiochemotherapy (RCT) and indication for surgical treatment. To estimate the number of samples required, statistical calculations were performed assuming an OSCC prevalence of 50% and an alpha of 5%. These calculations indicated that with a sample number of 80, a SRH sensitivity of 80% can be estimated with a 95% CI of 75–90%. The 80 tissue specimens were taken from 8 OSCC patients who were included into the study between May and July 2022. Directly after surgical resection, the native (not formalin-fixed) tissue specimens were transferred to the Institute for Surgical Pathology (ISP) at the University Medical Center Freiburg. Upon arrival and before tissue sampling, all specimens were macroscopically examined by a pathologist. Subsequently, tissue samples with a maximum size of 0.4 × 0.4 × 0.2 cm were taken from macroscopically tumor suspicious specimen areas and from specimen areas that appeared macroscopically tumor-free, aiming for an overall ratio of 50:50 of tumor-positive to tumor-free samples. To ensure that standard diagnostic histopathologic evaluation was not compromised by this procedure, all specimens were taken by experienced pathologists.

### Stimulated Raman histology imaging

**Fig. 1:**
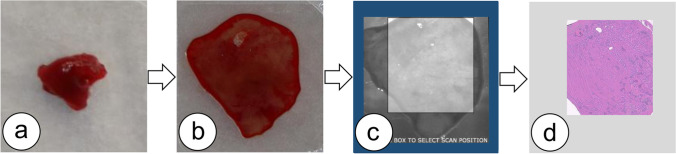
Process of SRH acquisition. Fresh tissue samples (**a**) were placed onto a custom microscope slide and compressed with a glass coverslip applying gentle manual pressure (**b**). Once loaded into the NIO Laser Imaging System, the selection window was set (**c**) and acquisition of SRH images (**d**) was initiated

Every tissue specimen was placed onto a custom microscope slide composed of a plastic object carrier, a spacer, and a glass coverslip. Through gentle application of manual pressure, the specimen was then compressed to the maximum height of the spacer (230 μm; Fig. 1B) and subsequently loaded into the NIO Laser Imaging System. No further processing, staining, or labeling was required. Within the NIO Laser Imaging System software, the rectangular selection window was manually set to cover a maximal area of the tissue specimen (Fig. 1C) and SRH image acquisition was initiated.

Stimulated Raman histology acquisition was performed as previously reported [10, 12]. In short, Raman shifts corresponding to CH2 bonds and CH3 bonds were applied to create the raw data by performing multiple line scans with a width of 1000 pixels (pixel size 467 nm) at a depth of 10 μm below the coverslip. These images are automatically stitched generating 4–5-μm-thick virtual sections, and subsequently converted into SRH images (“pseudo H&E images,” which are reminiscent of conventional H&E images) by subtraction and color assignment via a proprietary lookup table included in the software of the device. Following SRH image acquisition, H&E-stained frozen sections and formalin-fixed paraffin-embedded (FFPE) sections were prepared from each tissue sample. As H&E-stained frozen sections represent the gold standard for intraoperative histopathologic diagnosis in oral squamous cell carcinoma, these sections were used to evaluate SRH performance.

### Preparation of H&E-stained frozen sections

After acquisition of SRH images, samples were removed from the custom microscope slide and placed on a metal tissue disc in a gel-like embedding medium (OCT) in the same orientation as on the custom microscope slide. After rapid freezing to − 25 °C, 4–10-µm-thick cryo sections were generated using the microtome portion of the cryostat (Leica CM1950). Subsequently, the sections were picked up on a glass slide, H&E stained (using the Leica ST4020 Linear Stainer), and covered with a coverslip. When processing frozen tissue samples, the samples are often only partially cut in the first sections due to their positioning on the metal tissue disc. In the present study, these first sections were discarded and the first section representing the sample in its entirety was used for further analysis. Thus, the sections evaluated were generated at a “depth” of approximately 100 µm.

### Histopathological evaluation

**Fig. 2:**
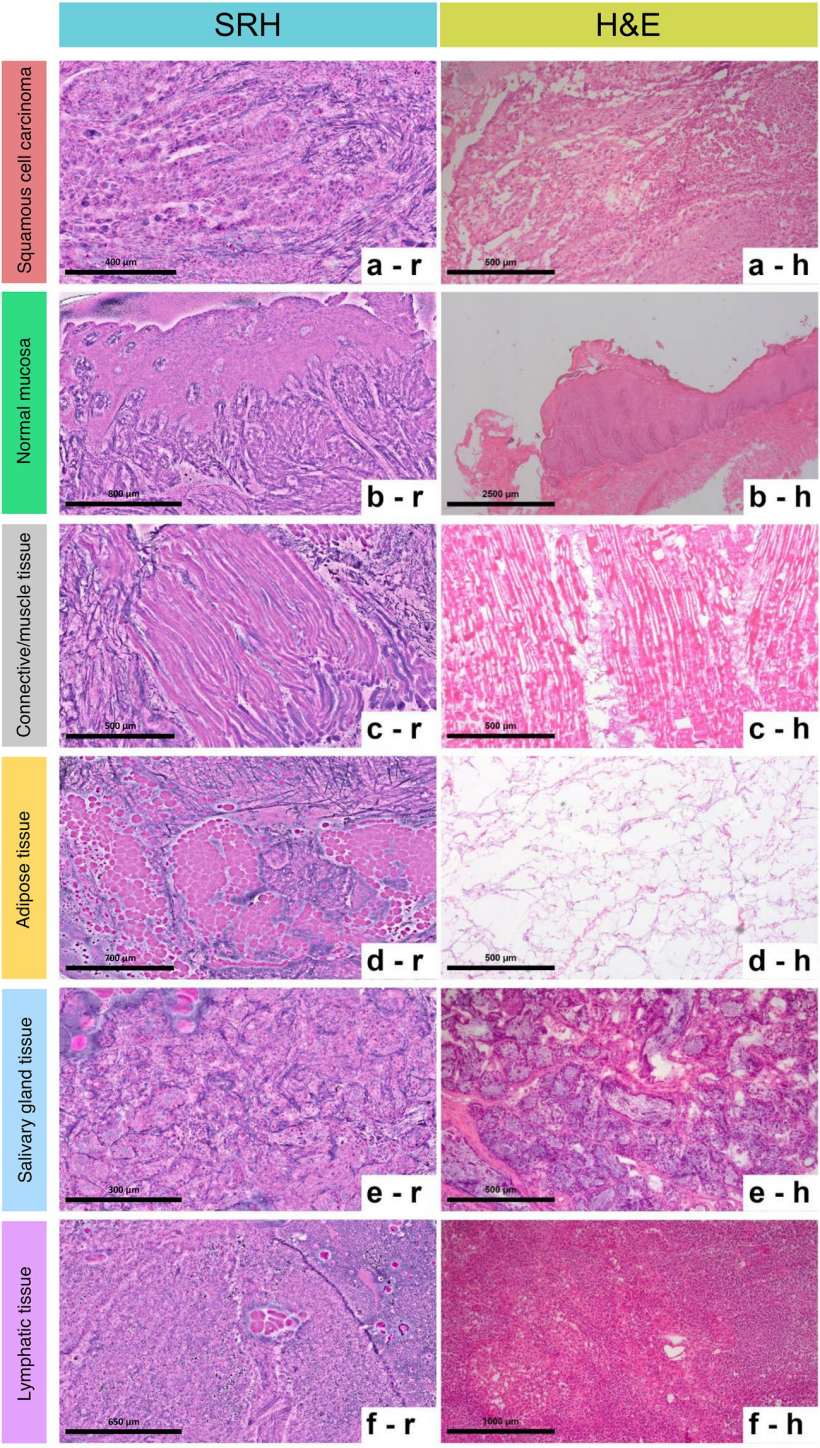
Overview of the tissue types evaluated in the present study. Representation in stimulated Raman histology images (left column) and H&E-stained frozen sections (right column).

**Fig. 3:**
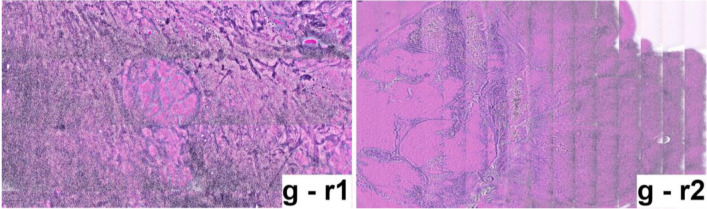
“Reduced image quality” as observed in SRH images

Representative SRH images including the study relevant tissue types (squamous cell carcinoma [Fig. 2a], normal mucosa [Fig. 2b], connective tissue [Fig. 2c], muscle tissue [Fig. 2c], adipose tissue [Fig. 2d], salivary gland tissue [Fig. 2e], lymphatic tissue and inflammatory cells [Fig. 2f]) were used to train the investigators. After a period of 4 weeks, the complete cohort was analyzed for the presence or absence of the abovementioned tissue types by a pathologist and an oral and maxillofacial surgeon. In cases where the two observers did not agree on the diagnosis, a third observer (board-certified pathologist) was consulted. In addition to the predefined tissue types, “reduced image quality” resulting e.g. from focusing problems and tissue artifacts (Fig. 3) was added as a further parameter during analysis.

After SRH image analyses, the H&E-stained frozen sections were analyzed for the presence respectively absence of the abovementioned tissue types by two pathologists, who were blinded to the results of the corresponding SRH images.

### Statistical analysis

For all images/sections (SRH images, H&E frozen sections), the presence or absence of the abovementioned tissue types (squamous cell carcinoma, normal mucosa, connective tissue, muscle tissue, adipose tissue, salivary gland tissue, lymphatic tissue, and inflammatory cells) was recorded in an Excel spreadsheet (MS Excel, Microsoft, Redmond, Washington). The primary outcome parameter for statistical evaluation was detectability of OSCC; the possibility to subclassify non-neoplastic tissue was evaluated as a secondary outcome parameter.

Cohen’s kappa was applied for quantitative evaluation of the agreement between H&E-stained frozen sections and SRH images. To quantify the accuracy of SRH in detecting the above mentioned tissue types, sensitivity, specificity, positive predictive value (PPV), and negative predictive value (NPV) as well as area under the receiver operating characteristic curve (AUC) were computed.

To evaluate the impact of reduced image quality on the detectability of the different tissue types, logistic regression models were applied. All statistical analyses were performed using the statistical software STATA (Version 17.0, StataCorp LLC, College Station, TX, USA). The level of statistical significance was set to 0.05.

## Results

A total of 80 tissue samples were included into the present study. The samples were obtained from eight OSCC specimens. Thereof — based on conventional H&E staining — 36 tissue samples were classified as OSCC. In 4 specimens, definitive exclusion of OSCC was not possible in SRH imaging, and these samples were included in the statistical analysis as tumor-positive cases. The rationale for this approach was that in a case where the presence of OSCC could not be excluded in intraoperative diagnosis, the area in question would most likely be classified as tumor positive and further resection would be performed.

Regarding the primary outcome parameter (detectability of OSCC, which is the pivotal question of intraoperative histopathologic diagnosis in the surgical treatment of head and neck cancer), high agreement (kappa: 0.880) and high accuracy (sensitivity: 100%; specificity: 90.91%; PPV: 90.00%; NPV: 100%; AUC: 0.954) were demonstrated when comparing SRH imaging to conventional H&E-stained frozen sections. Features enabling the identification of OSCC in the SRH images included standard cytological (e.g., pleomorphic hyperchromatic nuclei, irregularly dispersed chromatin, prominent nucleoli, and keratinisation) and histological/architectural (e.g., stratification disorder, infiltrative growth) changes. Detailed evaluation of the cases in which detection of OSCC by SRH was not possible revealed that this was (mainly) due to blurred resolution and superficial SRH “cutting”/admission.

**Fig. 4:**
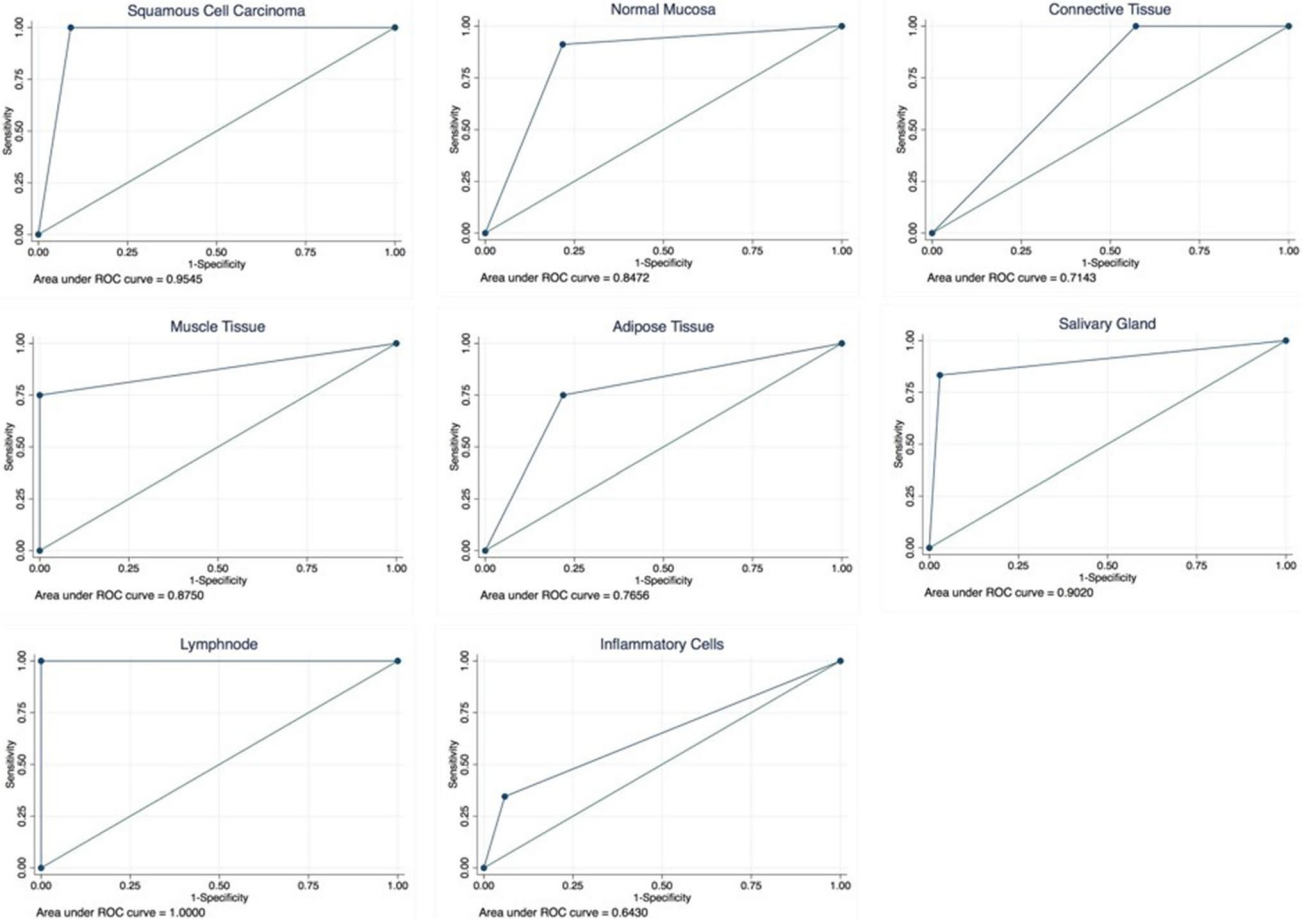
Receiver operating curves and areas under the receiver operating characteristic curve. Individual figures show the performance of SRH analysis in detecting the different tissue types included in this study, considering H&E-stained frozen sections as “gold standard”

Regarding the secondary outcome parameter (sub-classification of non-neoplastic tissue), a representation of SRH reminiscent of conventional H&E was found for muscle tissue and salivary glands (Fig. 2c/e). In other tissues like connective tissue and adipose tissue however, notable differences considering histological architecture were observed (Fig. 2d/f). This was reflected in the statistical analyses, where highest agreement between SRH and frozen section–based histology with kappa > 0.8 was found for muscle tissue (Fig. 2c) and salivary glands (Fig. 2e). A detailed overview for sensitivity, specificity, PPV, NPV, and kappa (with respect to the detectability of the tissue types included in this study using SRH) is provided in Table 1 and Fig. 4.

**Table 1:** Overview of sensitivity, specificity, PPV, NPV, and kappa

	H&E frozen sections		Stimulated Raman histology		Sensitivity (95% CI)	Specificity (95% CI)	PPV (95% CI)	NPV (95% CI)	Kappa
	Detectable	Not detectable	Detectable	Not detectable					
Squamous cell carcinoma	36	44	36*	40*	100.00% (100.00–100.00%)	90.91% (84.61–97.21%)	90.00% (83.43–96.57%)	100.00% (100.00–100.00%)	0.8804
Normal mucosa	34	46	41	39	91.18% (84.96–97.39%)	78.26% (69.22–87.30%)	75.61% (66.20–85.02%)	92.31% (86.47–98.15%)	0.6762
Connective tissue	73	7	77	3	100.00% (100.00–100.00%)	42.86% (32.01–53.70%)	94.81% (89.94–99.67%)	100.00% (100.00–100.00%)	0.5778
Muscle tissue	8	72	6	74	75.00% (65.51–84.49%)	100.00% (100.00–100.00%)	100.00% (100.00–100.00%)	97.30% (93.74–100.00%)	0.8437
Adipose tissue	48	32	43	37	75.00% (65.51–84.49%)	78.12% (69.07–87.18%)	83.72% (75.63–91.81%)	67.57% (57.31–77.83%)	0.5178
Salivary gland	12	68	12	68	83.33% (75.17–91.50%)	97.06% (93.36–100.00%)	83.33% (75.17–91.50%)	97.06% (93.36–100.00%)	0.8039
Lymphnode	3	77	3	77	100.00% (100.00–100.00%)	100.00% (100.00–100.00%)	100.00% (100.00–100.00%)	100.00% (100.00–100.00%)	1.000
Inflammatory cells	29	51	13	67	34.48% (24.07–44.90%)	94.12% (88.96–99.27%)	76.92% (67.69–86.16%)	71.64% (61.76–81.52%)	0.3246

^*^In 4 cases definitive exclusion of tumor not possible

Reduced image quality was observed in 17 of the 80 SRH images, especially in larger samples. Evaluating the impact of this factor on the detectability of the different tissue types in SRH, it could be shown that reduced image quality had an impact on the detectability of the different tissue types, with an odds ratio (OR) ranging from 0.30 for salivary glands to 1.77 for adipose tissue. However, none of these effects were statistically significant.

## Discussion

In the present prospective study, the agreement between SRH and conventional H&E-stained frozen sections in detecting tumor tissue in samples obtained from OSCC patients and in further differentiating non-neoplastic tissue was evaluated. Conventional frozen section diagnosis is widely available, has a long history of use in surgical pathology, and has been proven a reliable method for histological diagnostics [13]. The diagnoses derived from frozen sections directly influence the surgical procedure, with further resection performed in case of tumor-positive margins. The principles of the technique applied for frozen section analysis were first described at the beginning of the twentieth century [14]. Whereas technological developments have been made, the basic features are still unchanged today with sectioning and staining making this technique time and labor intensive and requiring a well-equipped histopathology laboratory with experienced technical assistants.

SRH was introduced in 2017 and offers the ability to create digital images reminiscent of conventional hematoxylin and eosin (H&E)–stained frozen sections without the need for sectioning and staining [10]. In the histopathological evaluation of brain tumors, SRH has already been shown to provide excellent agreement with conventional H&E staining [10, 11]. The focus of SRH development was in highlighting key histoarchitectural features of central nervous system (CNS) tumors [10]. Due to the different composition of normal CNS and tumor tissue, the higher cellularity in CNS tumors leads to a higher contrast (compared to the non-tumor tissue) and to a better resolution, respectively a better discrimination between the two diagnoses (tumor/non tumor) [15]. In contrast to CNS tissue respectively CNS tumors, within OSCC the cellular composition is more heterogeneous and the cellularity is strongly increased [16], which complicates the interpretation of the respective tissue types.

However, some previous investigations have demonstrated the potential of Raman scattering to generate (pseudo)microscopic images of tissue samples obtained from OSCC patients. In 2015, Cals et al. published a report on point-by-point Raman mapping to generate pseudo-color Raman images from unstained thin tissue sections, which were clearly linked to the histopathological evaluation of the same section after H&E staining [17]. In 2017, Hoesli et al. published a report on coherent Raman scattering microscopy for the evaluation of head and neck carcinoma, using a method that can be considered a precursor to the technology evaluated in the present study [18]. In their study, Hoesli et al. reported a sensitivity of 88.1% and specificity of 95.2% in distinguishing between neoplastic and non-neoplastic tissues. These values are similar to the results obtained with SRH in the present investigation, whereas our study also demonstrated the applicability of SRH for sub-classification of non-neoplastic tissue and thus beyond the sole binary differentiation into neoplastic and non-neoplastic.

In the present study, the performance of SRH was evaluated by a comparison with H&E-stained frozen sections, as this technique represents the gold standard for intraoperative histopathologic diagnosis in oral squamous cell carcinoma. In this context, it has to be considered that frozen sections have their own limitations when compared to the histopathological gold standard of H&E-stained FFPE sections. However, regarding the diagnostic accuracy, the discrepancy rate between frozen sections and corresponding permanent sections is reported to be low [19] and this could be confirmed in the present study, where a discrepancy was found for only one specimen (no tumor in frozen section H&E, tumor detected in FFPE section).

Regarding the time required for histological diagnosis, with SRH and conventional frozen sections, the time required depends on different factors. In frozen section diagnosis, the duration is strongly dependent on the number of tissue samples sent in simultaneously. Data derived from the Institute for Surgical Pathology at the University Medical Center Freiburg in 2022 demonstrated that on average, 6.3 min were needed per specimen (variation between 2.4 and 16.7 min). With SRH, the duration is strongly dependent on the size of the tissue sample respectively the scanning window. In the present study, to ease comparison with H&E sections, large scanning windows were chosen to cover a maximum area of the sample, resulting in a mean scanning duration of 18.27 min. However, smaller scans can be acquired much faster (≈ 3 min for 7 mm^2^). Thus, reducing the size of the scanning window and possibly acquiring additional scans at different positions of the sample in case of inhomogeneous tissues could reduce the time required for SRH-based diagnosis in a clinical workflow. Moreover, it has to be considered that SRH imaging can be performed on site in the operating room, eliminating the time-consuming transfer of samples from the operating room to a histopathology laboratory.

From a technical point of view, the tissue to be examined must be (uniformly) compressible in order to create the squash preparation. In the present study, SRH scans with a thickness of 4–5 µm were generated at a depth of 10 µm. The specimens were then transferred to metal tissue disks for frozen section preparation, taking care not to change their orientation. Frozen sections of 4–10 µm were then prepared at a “depth” of approximately 100 μm; thus, SRH did not analyze the exact same layer of the specimen compared to the H&E-stained frozen sections. However, considering the size of the structures evaluated, it can be assumed that this did not lead to a major bias in the evaluation of the different tissue types.

Regarding the interpretation of the images, compared to conventional H&E staining, the resolution of SRH can be considered relatively low. As a result, it may be difficult to distinguish between tumor tissue and normal, tumor-free tissue. Moreover, the identification or interpretation of precursor lesions (low- and high-grade intraepithelial neoplasia as well as carcinoma in situ) is not possible with the currently available SRH resolution and these aspects may have contributed to the inability to make a definitive diagnosis regarding the presence of OSCC with SRH in 4 cases. In addition, the maximum scan area of the device is currently limited to approximately 25 mm^2^, and large specimens may introduce artifacts that affect image quality, as the autofocus is determined at the center of the specimen, with differences of thickness in large specimens resulting in focusing on areas that do not contain a sufficient amount of tissue. In the future, this problem will likely be minimized through the use of updated software that allows multi-scanning with repeated focusing. In addition, image quality could be further improved by fine-tuning the microscope and adjusting the staining algorithm specifically for use in oral/head and neck tissues.

In general, with regard to image interpretation, it should be noted that pathologists need to review several thousand slides to gain a certain level of experience. Since only a limited number of centers currently have access to SRH-based histology, the potential technology shift from frozen section/conventional H&E-based histology to SRH is likely to take a long time. In the present study, observers were blinded to the diagnosis of each specimen as determined by H&E-stained frozen sections (normal tissue/tumor tissue/lymph nodes etc.). However, all patients included in the study had a diagnosis of oral squamous cell carcinoma. This fact likely introduced some bias, as observers were not confronted with samples derived from patients not diagnosed with OSCC or random findings, which might have contributed to the high sensitivities and specificities, as well as the high negative and positive predictive values of this study.

## Conclusion

The present study has demonstrated the potential of SRH for the analysis of oral squamous cell carcinoma and the sub-classification of tumor-free tissue from the head and neck region; however, this technology is still in its infancy in this field. Appropriate processing protocols, preferably tissue-specific, need to be established for successful implementation of SRH into a clinicopathologic workflow. In addition, pathologists need to have more contact with SRH to gain experience. Therefore, a combination of conventional histological methods and SRH could be beneficial in introducing SRH into a clinicopathologic workflow. In this context, the inherently digital format of SRH images provides an ideal basis for artificial intelligence–based analysis of the data. This approach has already yielded very promising results in the interpretation of SRH images in the field of neurosurgery [20] and is now being evaluated by our group to take another step towards a fully digitized assessment of surgical margins in patients with head and neck tumors.

## Data Availability

The datasets used and analyzed during this study are available from the corresponding author on reasonable request.
